# The Strength of a Story: Partnering With a Community Organization to Destigmatize Substance Use Disorder

**DOI:** 10.15766/mep_2374-8265.11487

**Published:** 2025-01-24

**Authors:** Noah Sorkow, Cameron Lauf, Stephen H. Berns

**Affiliations:** 1 Fourth-Year Medical Student, Robert Larner, M.D., College of Medicine at the University of Vermont; 2 Executive Director, Turning Point Center of Chittenden County; 3 Associate Professor, Division of Palliative Medicine, Department of Family Medicine, Robert Larner, M.D., College of Medicine at the University of Vermont

**Keywords:** Community Partnerships, Patient Narratives, Small-Group Discussions, Substance Use Disorder, Case-Based Learning, Pain Medicine, Reflection/Narrative Medicine, Substance Abuse/Addiction, Community-Engaged Learning, Community Engagement

## Abstract

**Introduction:**

Stigmatizing attitudes held by health care professionals against individuals with substance use disorder (SUD) result in worse clinical outcomes. Story-listening has been shown to help mitigate bias for medical trainees. We created a narrative-based small-group facilitated discussion between medical students and an individual in recovery from SUD through a direct partnership with a community peer-recovery organization.

**Methods:**

All session materials were formulated in direct partnership with the community organization. After completing prework, second-year medical students engaged in a 1.5-hour facilitated discussion with a community member in recovery and one attending physician preceptor. Student perceptions of the session and SUD were evaluated using open-ended and Likert-scale questions through an online survey. Community members engaged in their own postsession debrief.

**Results:**

One hundred twenty-four second-year medical students, 16 attending physician preceptors, and 10 community members in recovery participated in the session. Students agreed or strongly agreed that they appreciated the session format (92%), that they learned something new about SUD (83%), and that the session was applicable to their future career (92%). Students appreciated the small-group format and hearing someone's lived experience/perspective. Community members expressed how the session helped humanize health care providers and had interest in participating in future sessions.

**Discussion:**

Working in direct partnership with a local community organization to create an intimate narrative-based educational experience for medical students is feasible, appreciated by medical student participants, and mutually beneficial to community members and can facilitate positive changes in student knowledge and, potentially, bias regarding SUD.

## Educational Objectives

By the end of this activity, learners will be able to:

1. Recognize how stigmas and a health care provider's interactions can both help and harm those with substance use disorder.
2. Practice perspective taking to better understand the disease of addiction.
3. Engage in compassionate listening and self-reflect on their own biases, assumptions, and privileges within the context of caring for persons with substance use disorder.
4. Name inclusive strategies such as language choice in advocating for a patient with substance use disorder.

## Introduction

In late 2022, the Centers for Disease Control and Prevention reported that the average life expectancy in the United States fell for the second year in a row.^1^ The report suggested that the primary drivers for this drop in life expectancy were COVID-19 and drug overdoses, most notably, synthetic opioids. Rates of substance use for Americans of all genders, races, and ethnicities have continued to rise in the past few years,^2^ particularly due to COVID-19, when increased rates of overdose were related to depression, stress, financial strains, and isolation.^3^ The continued rise of opioid-related deaths and substance use disorder (SUD) has impacted families, communities, and the health system, costing 442 billion dollars annually.^4^ There are complex and deeply rooted reasons for the rise in substance use and, specifically, the opioid epidemic, including stigma for those with opioid use disorder (OUD). Stigma is defined as a process where people with a particular social identity are labeled, stereotyped, and devalued, leading to discriminatory behavior against them.^5,6^ Public stigma has been shown to be a significant barrier to SUD treatment, leading to lower rates of treatment seeking and poorer treatment outcomes.^7^ A participant in a focus group around SUD stigma shared, “My son would not reach out for treatment… [He] just didn't want to be thought of that way…. I tried to get him to consider treatment and he would not because of the stigma.”^8^ Studies have found that stigma may also lead to decreased self-esteem, fear of punishment, and worsening overall physical and mental health.^9^ Health care workers still have stigmatizing attitudes and biases towards persons with SUD,^10–12^ leading to worse clinical outcomes, including delay in treatments for SUD and effective pain management.^13,14^ Because of this health care crisis, there has been a call to better educate our trainees about SUDs with an emphasis on mitigating bias.^15–17^

Several educational methods have been shown to reduce stigma among trainees, including clinical experiences,^18^ patient panels,^19,20^ and online trainings.^21^ Narrative and contact-based education has been shown to have a significant impact on building compassion, increasing empathy, and reducing emotional exhaustion and stigma.^22,23^ For OUD, a recent study that combined visual campaigns about nonstigmatizing language and a narrative vignette featuring the voices of people with OUD had more impact than visual campaigns alone in reducing stigma.^24^ Interestingly, Mort, Díaz, and Beverly demonstrated that medical students who experienced a five-member panel of opioid content experts and an individual in recovery had no difference in OUD stigma scores compared to students who attended lectures on OUD treatment.^25^ They hypothesized that the narrative presented in the panel concentrated more on career-related stories as opposed to personal experiences.

Involving community partners to help shape educational activities with a focus on personal narratives can help provide perspectives that are unique and impactful. When instructing trainees on the social determinants of health, including community organizations has been recommended.^26^ It is also apparent that involving community organizations in medical education is appreciated by students and is mutually beneficial to the community partner in helping them advance their mission.^27,28^ There is, however, little evidence on whether a direct community partnership and hearing patient stories in an intimate small-group setting would be feasible, be appreciated by learners, and change the perceptions of both the students and the guests sharing their narratives. We at the Robert Larner, M.D., College of Medicine at the University of Vermont (LCOM) partnered with a community organization for persons in recovery from SUD to help guide the content of an intimate storytelling experience. Together, our aim was to assess the feasibility and acceptability of a workshop focusing on personal narratives of individuals with SUD, with a secondary aim of increasing awareness of and changing perceptions about SUD in medical trainees.

## Methods

### Curriculum

The curriculum at LCOM included a longitudinal course called Professionalism, Communication, and Reflection (PCR) that aimed to develop professionalism, interpersonal skills/communication, and self-assessment/reflection. This course was delivered to medical students in a consistent process-oriented discussion group with a physician preceptor from first year through the end of third year. Physician preceptors represented a variety of specialties and were all provided with facilitation skills training prior to the start of the course (Appendix A). PCR had five sequential themes building off one another and tracking alongside clinical exposure. Our session was placed in the Advocacy in Medicine theme completed by preclinical second-year medical students. This timing allowed students to be exposed to perspectives and narratives regarding addiction and SUD prior to their exposure to the hidden curriculum of the clinical environment.

Students shared that the previous year's Substance Abuse and Addiction session, which had consisted of reading and discussion, felt disengaging and distant from the actual opioid crisis in Vermont. To improve upon the past session, we partnered with the Turning Point Center of Chittenden County—a peer-recovery support organization—to cocreate a new session that would deliver local community perspective and lived experiences.

Through this partnership, we developed a series of 10 questions meant to elicit a speaker's experience with SUD and, specifically, any instances in which they had experienced bias on behalf of health care providers (Appendix B). These questions were refined based on the input of second-year medical students to assess for any student-perspective gaps. The questions were used to create a facilitation guide that was distributed in advance to students, faculty, and community guest speakers (Appendix B). The required set of 10 questions listed in the facilitation guide covered introductions, the guest's substance use narrative, and stigma/bias they had experienced while interfacing with health care. This helped ensure standardization across all PCR groups.

#### Prework

Second-year medical students were given two prework assignments (a TED Talk and a *New York Times* article) to introduce the social factors confounding the development and treatment of SUD. Prework did not cover the pathophysiology of addiction and treatment as this was covered elsewhere in the medical school curriculum. The TED Talk, *Everything You Think You Know About Addiction Is Wrong,* detailed the socioeconomic and psychosocial factors behind addiction.^29^ The *New York Times* article, “It's Misleading to Call Addiction a Disease,” was an op-ed written by a physician with SUD that argued whether or not addiction should be considered a disease.^30^ This prework was selected directly with representatives from the Turning Point Center. As a substitute for these prereadings, we would recommend an article entitled “Language Matters: It Is Time We Change How We Talk About Addiction and Its Treatment,” which details evolving perceptions on discussing SUDs.^31^

#### Session structure

Each PCR group (six to eight students) and its physician preceptor were paired with a guest speaker in recovery from SUD from the Turning Point Center. Speakers were provided with a list of the students in each PCR group a week before the session to avoid personal or privacy conflicts. In the hour before to the sessions, all guest speakers underwent an in-person briefing covering an introduction to PCR, where the students were in their medical training, and the general format of the session.

The 90-minute session was student led, with a physician preceptor/facilitator as a guide to ensure quality, consistency, and adherence to the written guide (Appendix B). Each session began with a 10-minute warm-up exercise where students brainstormed and shared reasons why someone in recovery and someone actively using might feel uncomfortable seeing a physician. Afterwards, the facilitator reminded the students of their community agreement, including confidentiality. The session then transitioned to question and answer with the Turning Point Center guest using the session guide. After the required questions had been asked, the session was opened for students and the physician preceptor to explore any additional questions about SUD with their guest. The speaker then left, and the session closed with the group sharing their takeaways.

#### Postsession debrief with guest speakers

A separate debrief was held for the guests after the session. This was a semistructured group session facilitated by the course director while the principal investigator took notes. Guests were asked what they felt had gone well, what they would like to change, and if anything came up for them during their session. After these questions, the debrief was opened to any other additional comments and concluded with gratitude for the guests.

### Evaluation

A survey was developed in direct collaboration with the Turning Point Center to assess feasibility of future iterations of this session and whether learning objectives had been met. An anonymous REDCap survey was distributed through email to the second-year medical student class. The survey featured 10 questions total, including free-response items, 5-point Likert-scale items (1 = *strongly disagree,* 5 = *strongly agree*), and an optional space for additional comments. Questions were aimed at identifying whether students appreciated the session format, if they had learned new information, if their perceptions had changed, and what they liked/disliked about the session (Appendix C). Survey responses were gathered and tabulated using Microsoft Excel.

The free-response questions were analyzed using a qualitative review of the commentary to identify and report common themes among responses. Both principal investigators independently reviewed responses and coded them to one or multiple themes. After this, the coded themes identified by each investigator were cross-compared to identify which of them were agreed upon. A descriptive analysis was done on each of the seven Likert-scale questions. Themes were also identified from the debrief sessions with community members through a discussion between the community partner lead and the principal investigators.

Feasibility was defined as whether or not we were able to successfully identify a community organization that would be willing to participate, form a partnership, design our facilitation guide with community input, recruit a sufficient number of community partners to engage with each group of students, and have at least 90% student participation. As such, data were collected on student attendance and numbers of community guest speakers who participated.

## Results

### Participants

All 124 second-year medical students from LCOM attended the session, along with 16 physician preceptors. Ten individuals in recovery recruited by the Turning Point Center participated, eight of whom participated in both sessions, allowing for one guest per PCR group. All guest speakers attended the pre- and postsession debriefs.

### Feedback From Community Guests

Overall, there was consensus that guests enjoyed participating in this program, although it made three of them nervous. The individuals who were nervous attributed this feeling to social anxiety and fears of public speaking. They reported that the anxiety diminished after interacting with the medical students and noticing that the students were interested in this topic. Multiple community members cited that this interaction humanized medical professionals in their eyes. For example, one community member in recovery stated that she was “surprised” by how easily she got along with the students and that she could see these individuals as future friends—a feeling she had never previously associated with medical professionals. Interestingly, one community member stated that participating in this session provided her insight into why her primary care provider asked her certain health screening questions during routine visits. The guest speakers expressed their desire to continue to participate in future iterations of this program, to help refine the prelearning materials to emphasize the socioeconomic drivers of SUD more adequately, and to further decrease the formality of the session to make the content more approachable to students who might be uncomfortable with this sensitive subject.

### Feedback From Students

A postsession RedCAP survey was distributed to all 124 student attendees; the response rate was 45%.

**Figure 1.**
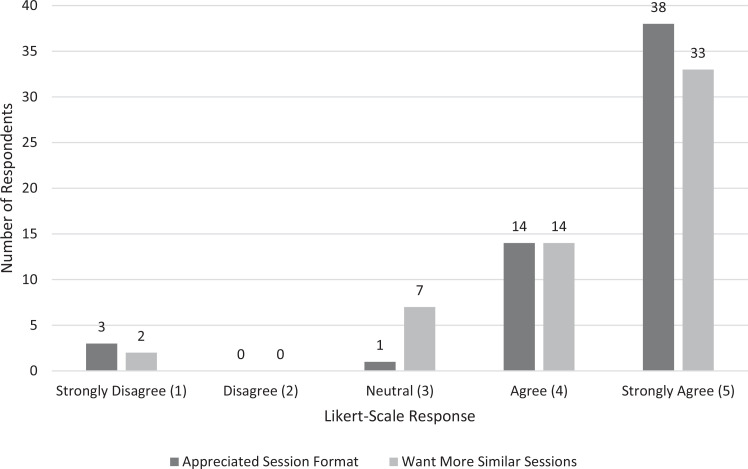
Numbers of students who self-rated on a 5-point Likert scale their appreciation for the session format and their desire to have similar sessions in the future.

The first two survey questions pertained to student opinions of the session's format (i.e., a facilitated question-and-answer session with a community member in recovery) as shown in Figure 1. Ninety-two percent of respondents agreed or strongly agreed with the statement that they appreciated the session format, with 67% strongly agreeing. Eighty-three percent of respondents agreed or strongly agreed with the statement that they wanted more sessions similar to this experience in the future.

**Figure 2.**
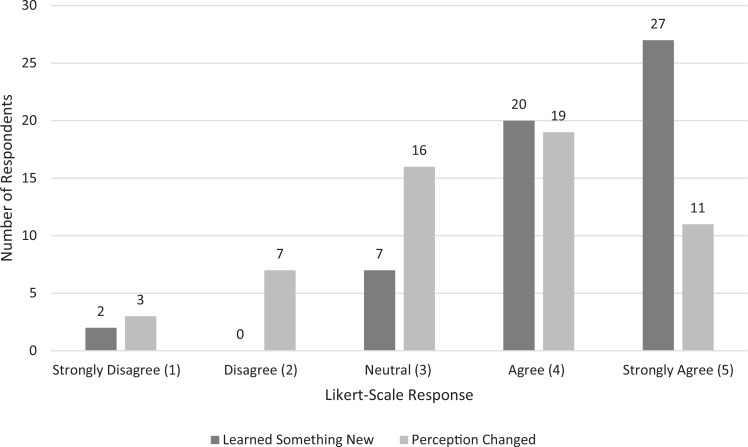
Numbers of students who self-rated on a 5-point Likert scale their changes in knowledge and perception.

The next two survey questions assessed students’ perceptions on a change in their knowledge about SUD and their own biases (Figure 2). Eighty-three percent of respondents agreed or strongly agreed that they learned something new about SUD because of the session. Fifty-three percent respondents agreed or strongly agreed that their perceptions about individuals with SUD were changed because of the session, while another 28% were neutral regarding their perception changing.

Ninety-two percent of students agreed or strongly agreed when asked about the session's applicability to their future career in medicine.

Seventy-six percent of students agreed or strongly agreed with the statement that they now recognized opportunities to get involved with volunteering and advocacy programs within the SUD community.

Answers to three free-response questions regarding what was appreciated about the session (Table 1) and what students wanted to improve upon (Table 2) are tabulated by theme, frequency of themes, and exemplary responses.

**Table 1.**
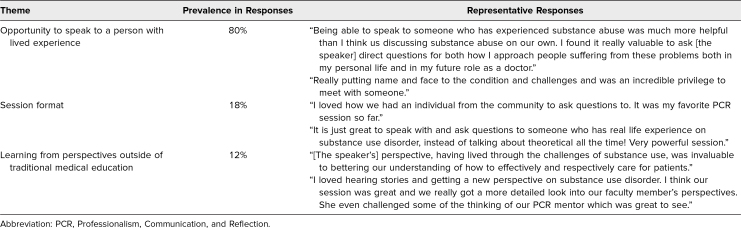
What Students Appreciated About the Session

**Table 2.**
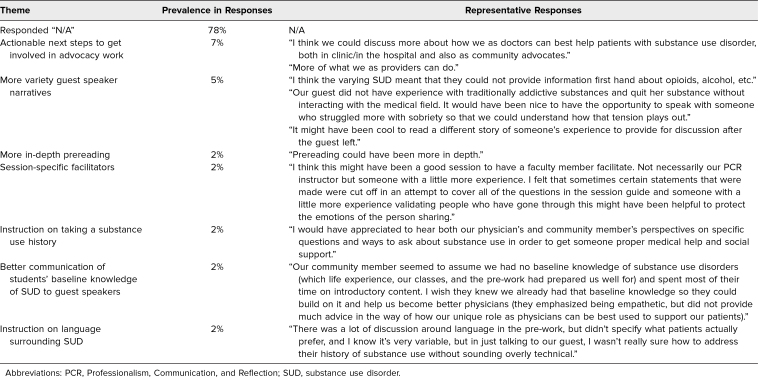
What Students Thought Was Missing From the Session

Only two students provided optional additional comments. The first student enjoyed the TED Talk provided as part of the prelearning materials. The second student thanked the team for working on improving PCR in the preclinical years.

## Discussion

Our curriculum on substance use and stigma successfully demonstrated a feasible model for collaborating with a community organization to ensure an intimate, story-listening experience for medical students. Our community partner successfully recruited a sufficient number of persons in recovery to accommodate each small group and directly collaborated in all steps of curriculum development. Next, students shared their strong appreciation for the intimate session format and ability to hear a person's lived experience. They shared that this allowed for discourse that challenged misconceptions and biases and that they gained new perspectives and insights into SUD. One student commented that it even changed the perspective of their physician preceptor. Students felt that this experience was applicable to their future no matter their desired specialty. Community members remarked that this was an overall positive experience in contrast to their past interactions with the health care system. The community guests shared that this experience humanized medical professionals and provided insight on why clinicians choose the questions they ask of their patient, for example. Community guests shared enthusiasm for wanting to repeat the session the following year. While not directly one of the aims of the curriculum, it became apparent that guest speakers developed a more positive opinion of health care providers through their participation in the session. We believe that the small-group guest experience over a patient panel discussion allowed for vulnerability from both students and guests and helped change perceptions mutually.

Our emphasis on sharing the narratives of community members, although greatly appreciated by most students, left some of them feeling a lack of concrete clinical guidance. This contrasts with the Mort, Díaz, and Beverly study, which emphasized professional stories.^25^ Furthermore, based on student feedback, it became clear that while this curriculum provided in-depth exposure to a person's lived experience, it came at the expense of diverse narratives. A panel format can better preserve variety in narratives but may sacrifice intimacy and engagement. Alternatively, an additional written or recorded narrative from another community member could be added to the session materials.

There was initial concern that the strong relationships within each PCR group would present a barrier to community guest speakers feeling welcome and comfortable in sharing their story. However, speakers remarked that they felt immediately welcomed into this setting.

Our activity has multiple limitations. First, it was conducted at a single site and in a state known for its smaller population and hub-and-spoke model for substance use treatment. These factors offered more favorable conditions for creating community partnerships. Additionally, our cohort of 124 students was logistically easier to accommodate with guest speakers while maintaining the small-group format. Larger schools may have to recruit more individuals from outside their immediate local community partners or utilize a virtual format if they have multiple campuses or distant community partners. As the primary endpoint was to establish feasibility for the session, a presurvey was not conducted in an attempt to reduce medical student survey fatigue. Because of this, we were unable to measure the magnitude or direction of perception and stigma with postsurveys alone. Third, only 45% of students completed the survey, which may not have been representative of the entire class perspective. This relatively low rate of survey completion can likely be attributed to delayed survey distribution after the date of the session. Finally, we must mention that the existing curricular framework for PCR was conducive to our implementation of these sessions. Specifically, resources at our disposal included preformed small groups of medical students, preestablished longitudinal intragroup relationships, dedicated time within the medical school curriculum, dedicated physical space, and preassigned faculty preceptors who had already received facilitation training. Given these resources, there are no plans to separate this session from the larger PCR curriculum. However, these resources are not unique to PCR, and this activity can be implemented as a stand-alone session by adding presession time for faculty and guests to meet and review the session guide and facilitation skills (Appendices A and B). For programs without longitudinal small groups, it would be beneficial to spend time at the beginning of the session to create and reinforce a community agreement.

To further improve the curriculum, we plan on increasing the diversity of narratives via video vignettes to be included in the prework, create dedicated faculty development time regarding SUD language and sensitivity, and reserve 10 minutes at the end of each session for specific conversation about what can be done from the provider perspective.

To better assess the impact of the future sessions, we plan on conducting pre- and postsession surveys of students using a validated instrument that measures explicit bias regarding SUD and distributing the surveys during the session. This would better assess the session's efficacy in regard to the stated learning objectives and improve response rates.

For students who desire further involvement with this community, we plan to partner with the Turning Point Center to create a volunteer event for the creation of substance use recovery kits and their distribution.

## Appendices


Faculty Facilitation Skills Handout.pdfSession Guide.docxPostsession Survey.docx
*All appendices are peer reviewed as integral parts of the Original Publication.*
