# Supplementary material for: The Strength of a Story: Partnering With a Community Organization to Destigmatize Substance Use Disorder
Source: MedEdPORTAL. 2025 Jan 24;21:11487. doi: 10.15766/mep_2374-8265.11487 (PMC11759220; doi:10.15766/mep_2374-8265.11487)
Supplement: Supplementary file 1 — Faculty Facilitation Skills Handout.pdfSession Guide.docxPostsession Survey.docx [file mep_2374-8265.11487-s001.zip › B. Session Guide.docx]

**Substance-Use Disorder and Addiction Session**

**Session Learning Objectives**

By the end of this session, students will be able to:

- Recognize how stigmas and a healthcare provider’s interactions can both help and harm those with substance-use disorder.
- Practice perspective taking to better understand the disease of addiction.
- Engage in compassionate listening and self-reflect on one’s own biases, assumptions, and privileges within the context of caring for persons with substance-use disorder.
- Name inclusive strategies in advocating for a patient with substance-use disorder such as language choice.

**Pre-Session Materials** (30 minutes on your own)

- Hari J. Everything You Think You Know About Addiction Is Wrong.; 2015. <https://www.ted.com/talks/johann_hari_everything_you_think_you_know_about_addiction_is_wrong?language=en>.
- Fisher C. Opinion | It’s Misleading to Call Addiction a Disease. Nytimes.com. <https://www.nytimes.com/2022/01/15/opinion/addiction-disease.html>. Published 2022.

**Session Materials**

- None

**Session Plan**

1. **Check-in** (10 minutes)
   - Check-in: How is class going for everybody?
2. **Brief Warm Up on Substance-Use Disorder** (20 min)
   - Think/Pair/Share – ask half the pairs answer question 1 and the half answer question 2. Have students pair up, discuss, and then share their thoughts with the group
     1. Question 1 – Why might someone in recovery from substance-use disorder be worried about going to see a doctor?

OR

- - 1. Question 2- Why might someone who is actively using be worried about going to see a doctor?
  - Quick Debrief—What is one new perspective that your pair had not thought about that you would like to share with the larger group?
  - Terms/Definitions
    1. **What is person-first language?** What are some examples of person-first language? **What is the difference between bias and prejudice**?

1. **Q&A with Community Member in Recovery** (50 min)
   - Each small group will sit down with an individual recruited by a community partner for a question-and-answer session on their experience being in recovery. **Before starting the questions and introductions, remind the group that everything that will be discussed during the session will remain confidential.**
     1. **Standardized questions to begin the Q&A**
        1. **Speaker Introductions**

- First name only
- What you currently do for work
- What is one thing that you are passionate about
  - - 1. **General questions about addiction**
- What factors led to you using?
- What factors led to you entering recovery?
- How many times did it take for you to find long-term recovery?
  - - 1. **Questions about bias**
- What is an example of a time a healthcare provider did something "right" in supporting or treating you or someone you know? What did they do?
- What is an example of a time a healthcare provider did not provide the support or inclusive treatment you needed or someone else needed? What did they do?
- What are some ways you found language around substance use with clinicians to be exclusive? What about inclusive?
- What is something you wished a doctor would have done or recognized when they interacted with you, or someone know when actively using? How would you want a healthcare provider to respond when you say that you are in recovery?
  - 1. **Floor is opened to any questions the group has that have not already been covered**

1. **Close** (10 minutes) Guest speakers should be thanked and then allowed to leave so that the small group can debrief on its own.
   - Ask the group to reflect on 3 questions and write down their answers
     1. **What surprised you about the session?**
     2. What is something you plan to take forward?
     3. What is something you are still wondering about?
   - Ask each member of the group to share one of their answers.

**Post-Session Materials**

- Dell Medical School Value-Based Health Care. “Reducing Stigma Education Tools (ReSET)”. University of Texas at Austin 2020.

<https://vbhc.dellmed.utexas.edu/courses/course-v1:ut+cn01+2020-21/course/>

- Maté G, Levine P. In The Realm Of Hungry Ghosts.
  - This is a great book on addiction written by a physician who worked with people with substance-use disorder in Vancouver, BC. It is a powerful tale of the many determinants that contribute to this disease.
- Brooklyn JR, Sigmon SC. Vermont Hub-and-Spoke Model of Care for Opioid Use Disorder: Development, Implementation, and Impact. J Addict Med. 2017;11(4):286-292. doi:10.1097/ADM.0000000000000310
  - This is an article published by a UVM physician who helped create a structure of care for opioid-use disorder that has since become a model nationwide.
- NIH Health Professions, [Words Matter](https://nida.nih.gov/nidamed-medical-health-professionals/health-professions-education/words-matter-terms-to-use-avoid-when-talking-about-addiction)

**How You Can Get Involved (**we suggest medical school, state, and national levels for involvement, examples that we used are provide below**)**

- LCOM/UVMMC
  - [Harm Reduction SIG](https://commons.med.uvm.edu/learning/gov/sigs/harm/default.aspx) – great educational and volunteer events – including Naloxone training
  - AHEC Addiction Medicine Elective – 4^th^ Year
  - [UVMMC Day One Program](https://www.uvmhealth.org/medcenter/conditions-and-treatments/substance-abuse)
- Vermont Level
  - [Turning Point Center of Chittenden County](https://turningpointcentervt.org/)- Peer-run recovery center operating out of downtown Burlington, as well as a peer recovery coach program in partnership with the UVMMC Emergency Department
  - [Howard Center](https://howardcenter.org/get-involved/)- Community organization that specializes in mental health and substance-use disorder
  - [Vermont Cares](https://vtcares.org/) – Provides needle exchange and other harm reduction services
- National
  - [National Institute on Drug Abuse](https://nida.nih.gov/nidamed-medical-health-professionals/health-professions-education) - Educational resources and more
  - [Student National Medical Association](https://snma.org/page/programs)

Session Tips

- ***Strongly encourage using bold questions****.*
- *The subject matter that the guest speakers will be speaking on is incredibly personal and often traumatic to discuss. It is a privilege and honor to be privy to these lived experiences****, be respectful and non-judgmental as one would be during any patient interaction.***
  - *Be mindful of the emotions and discomfort that this can provoke and take the opportunity to take care of your colleagues and guest.*
- *Substance-use disorder is extremely prevalent in our society. Please recognize that members of the group may have personal connections to this topic and may find some of the content triggering or distressing.* ***Remind members that it is okay to step away from the discussion and take time if they need.***
- *Encourage students to speak from personal experience with “I” statements.*
- *If there is a lull in the conversation, you can ask the group to reflect on their experiences round substance use either clinically or personally; you can bring the guest into the conversations by asking perspective or take on that interaction/experience.*
- *It can be helpful to take a moment and ask the group to pause or write down answers to help all compose thoughts.*
