# Supplementary material for: The Strength of a Story: Partnering With a Community Organization to Destigmatize Substance Use Disorder
Source: MedEdPORTAL. 2025 Jan 24;21:11487. doi: 10.15766/mep_2374-8265.11487 (PMC11759220; doi:10.15766/mep_2374-8265.11487)
Supplement: Supplementary file 1 — Faculty Facilitation Skills Handout.pdfSession Guide.docxPostsession Survey.docx [file mep_2374-8265.11487-s001.zip › C. Postsession Survey.docx]

# **Post-Session Survey: Substance-Use and Addiction**

Please complete the survey below. There are 10 questions, 9 of which are required. All except 1 required question follow a 5-point Likert scale that indicate how much you agree, or disagree, with the statement above. In total, this survey should take less than 5 minutes to complete.

1) I appreciated the session's format (Q&A with members

of the community).

[ ] Strongly Disagree [ ] Disagree [ ] Neutral [ ] Agree [ ] Strongly Agree

2) I would like to have more sessions like this one

moving forward in PCR.

[ ] Strongly Disagree [ ] Disagree [ ] Neutral [ ] Agree [ ] Strongly Agree

3) I learned something new about substance-use disorder

from this session.

[ ] Strongly Disagree [ ] Disagree [ ] Neutral [ ] Agree [ ] Strongly Agree

4) My perceptions of individuals with substance-use

disorder have changed after this session.

[ ] Strongly Disagree [ ] Disagree [ ] Neutral [ ] Agree [ ] Strongly Agree

5) The pre-work provided me with enough contextual

information to adequately participate in this session.

[ ] Strongly Disagree [ ] Disagree [ ] Neutral [ ] Agree [ ] Strongly Agree

6) This session was applicable to my future career in

medicine.

[ ] Strongly Disagree [ ] Disagree [ ] Neutral [ ] Agree [ ] Strongly Agree

7) I left this session with new knowledge on how I can

get involved in advocacy work regarding substance-use

disorder.

[ ] Strongly Disagree [ ] Disagree [ ] Neutral [ ] Agree [ ] Strongly Agree

8) What did you like about the session? ____________________________________________

9) What do you believe, if anything, was not adequately

covered during this session? (If no comments, please

put "N/A") ___________________________________________________________________

10) Any additional comments (not required) _________________________________________
