# Supplementary material for: The Strength of a Story: Partnering With a Community Organization to Destigmatize Substance Use Disorder
Source: MedEdPORTAL. 2025 Jan 24;21:11487. doi: 10.15766/mep_2374-8265.11487 (PMC11759220; doi:10.15766/mep_2374-8265.11487)
Supplement: Supplementary file 1 — Faculty Facilitation Skills Handout.pdfSession Guide.docxPostsession Survey.docx [file mep_2374-8265.11487-s001.zip › A. Faculty Facilitation Skills Handout.pdf]

This facilitation skills handout should be distributed to small group facilitators to review prior to the session. Can be reviewed independently or as a group (roughly 15 minutes).

Essential Facilitation Skills

DEFINITION OF A FACILITATOR

A facilitator is an individual who enables a group to work more effectively, to collaborate and achieve synergy, and support the group to do their best thinking and practice.

FACILITATOR GOALS

- Create an environment for effective communication and learning (brave space)
- Keep discussion focused on learning objectives
- Engage the entire group and foster a willingness to participate even after the session.
- Advance and deepen conversations, challenging the group to be self-reflective and work at their edge.

5 Skills Domains

There are 5 domains of skills for facilitating a small group:

1. **Prepare for the session**
2. **Create and maintain a brave space**
3. **Stay on task**
4. **Engage the group**
5. **Deepen conversations and learning**

The following document will review these key domains and the skills associated with them.

1. Prepare for the Session

Being prepared means you will confidently be able to handle unforeseen situations and adjust the pre-planned agenda more easily.

SKILL	DESCRIPTION	EXAMPLES & TIPS
**Review the materials**	It is important to go over the pre-session and session materials including the readings/media, learning objectives and session agenda.	*If there is a complicated activity, make sure you practice it so that it runs smoothly during the real session.*
**Plan with your co-facilitator**	If you will be facilitating with a co-facilitator (or preceptor). Make sure you align on your goals and negotiate roles.	*Questions to ask your cofacilitator:*<br><br>*"What is the most important goal you want to achieve for this session?"*<br><br>*"What parts do you want to lead? "Here is how I might ask for your help"*
**Strategize the session**	Decide on what engagement techniques you will use, what sections are musts, and how you will keep yourself on track	*Gather supplies that are needed including a timer. Print out handouts if needed.*

2. Create and Maintain a Brave Space

In recent years, we've seen the intentional shift within education to advocate for creating brave spaces over safe spaces. The best groups function in an environment where participants feel comfortable to share their views without judgement and also encourage dialogues stretch the participants, leading to some uncomfortable moments.

SKILL	DESCRIPTION	EXAMPLES & TIPS
**Model Vulnerability**	When the facilitator models vulnerability, participants are more likely to have the courage to also share their own vulnerabilities.	*Going First: "Let me share with one of the hardest moments for me when I was in middle school"*<br><br>*Saying I Don't Know: "That's a really good question. I am not sure how to answer that".*
**Start at Lower Stakes**	Warming up with a lower stakes activity before moving to the more complex can ensure more open discussions later in the session.<br><br>*Ice breakers* are a great way to build a sense of community and give participants the opportunity to be brave at lower stakes.<br><br>Participants may be nervous to share something in the larger group and so using subgroups (e.g. pairs) can a allow participants to ease into being vulnerable with the larger group.	*Using an Icebreaker: "As we are talking about facilitators today, can each of you go around and share who was your favorite teacher in elementary school and why?"*<br><br>*Pair and Share: "I will ask you all to pair off and answer who is your favorite teacher. Afterwards, I will ask each pair to share some common themes"*
**Encouragement**	Showing gratitude and praise can go a long way in promoting a brave sapce and reinforce risk taking.	*Gratitude: "Thank you so much for sharing that"*<br><br>*Precise Praise: "That's a really touching example of active listening"*

3. Stay on Task

As a facilitator your job is to balance both the objectives of the session and goals of the group. Helping the group stay focused can foster this balance.

SKILL	DESCRIPTION	EXAMPLES & TIPS
**Communicate Clear Instructions**	Effective facilitation is predicated on clarity. Providing instructions clearly and sequentially can explain the steps participants will take and allow them to stay focused on tasks.	*Be clear about time constraints.*<br><br>*If possible, show an example of the type of output you ask them to create.*<br><br>*Show instructions so they can refer to them as necessary. (For example, pre-write instructions on a flipchart paper or on a PowerPoint slide.)*
**Watch the Time**	To respect the outside obligations of the group, a session should always start on time and end time. Use a timer to help keep tabs on your progress. Call attention to time if the group is running behind.	*Using a timer or watch: There are some phone apps that can be useful in visualizing time (Time Timer app).*<br><br>*Calling attention to time: "I am noticing that we have 15 more minutes left to the session"*
**Interrupt if Needed**	If you feel like the group is going off task and/or you are running out of time, feel free to interrupt. You can still give the group control over their learning by asking permission to choose or decide how much time to spent on pieces of the session.	*Ask permission: "Would it be of if we talk about our own experiences with discrimination?"*<br><br>*Acknowledge and redirect: "I am hearing some really good comments, let's focus our attention on how spirituality affects patients who are having existential distress"*

4. Engage the Group

Group engagement is something the facilitator should be conscious of throughout the workshop and to make sure everyone contributes equally to the conversation.

SKILL	DESCRIPTION	EXAMPLES & TIPS
**Mix Interaction Types**	A healthy balance of different interaction types throughout your session will help participants to stay engaged.	*Think-Pair-Share: This activity promotes high level participation. It starts with participants having time to think about something individually, then discussing in pairs, and then sharing the ideas/conclusion with the entire group. This strategy is useful in the beginning of a session to energize the group.*<br><br>*Snowball: This method can be used to start discussions, develop new ideas or share something vulnerable. This starts with the facilitator asking a question. Each member of the group gets a paper and writes down their first thoughts to the question. When everyone has written their thoughts, each participant crumbles the paper and puts it into the middle of the table. Then, each participant grabs a crumbled piece of paper and reads the answers.*
**Encouraging Shared Airtime**	There may be times some participants are quiet and others are controlling the conversations. A facilitator should intervene to help balance the conversation either through warm calling, using round robins, and other techniques.<br><br>Warm call: Call on people who haven't yet said something but do it without calling out their name.	*Warm call: "Can I hear from someone who hasn't gone yet?"*<br><br>*Round Robin: "I will go around the room and ask everyone to share 1 idea they came up with"*<br><br>*Write it down first: Some people just need extra time. Asking people to write an answer down first enables everyone to think through a question carefully.*

5. Deepening Discussions & Learning

The goal for these conversations is not only for the group to feel comfortable to share and participant, it is also to feel challenged and walk away with a new idea or new perspective. Deepening the discussion can help push the group to the next

SKILL	DESCRIPTION	EXAMPLES & TIPS
**Clarifying**	Asking someone to clarify something can help to expand an idea or concept. It can also help turn something that is implied/vague to something clearer.	*"That's a really interesting… can you tell me more about what you mentioned?"*<br><br>*"Can you share what you meant by it's overwhelming?"*
**Building or Piggybacking**	Ask the group to build off of a comment to deepen it.	*"Would anyone want to add to Jeremiah's comment?"*
**Paraphrasing**	Paraphrasing means to express the same content that was just stated before but in your own words in order to check that both you and the others have the same understanding.	*"What I am hearing you say is that tracking non-verbal cues are really important to active listening"*
**Shifting Perspective**	If the group gets stuck at some point in the discussion or the discussion is one sided, try to shift the perspective and look at the problem from a different angle.	*"We talked about why this is a positive thing, why it could be a problem?"*<br><br>*"What might your parents or grandparents say about that?*
**Summarizing**	Repetition promotes understanding, and summarizing what has been discussed so far will help the group build upon the conclusions they have already made.	*"So we have talked about the Maslow's hierarchy of needs and how they can stress patients when they are missing. Let's now turn our focus on what we can do as physicians to support patients when the physical needs are missing".*

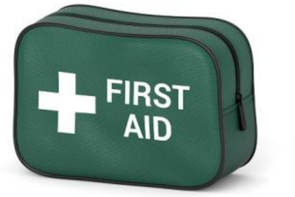

Facilitation First Aid Kit

CHALLENGE	EXPLANATION	SOLUTION
**Group has low energy**	Energy could be low at the end of the day or after long runs of the same activity.	• *Use an energizing activity as needed.*<br>• *Energizing activities can include Pair & Share, Snowball, or try to get them out of their seats.*
**Group is swayed by the facilitator**	A facilitator often has lots of power over the group even if they have no formal power role. This runs the risk of influencing a potential outcome of a discussion, thus detracting from safety.	• *Stay neutral. Hold off on providing your own opinions at the start of a discussion (you can still show vulnerability early though).*<br>• *Offer other perspectives if not being discussed.*
**Group is hard to read**	Sometimes groups may not share what is on their mind either because they feel unsafe or they are tired.	• *Checking in can determine the mood or general opinion of the group about a certain topic or point in the discussion.*<br>• *Asking for a "thumbs up- thumbs down" survey can be enough to get an impression.*
**Group discussion does not go as planned**	Usually, activities and discussions end up taking more time than initially planned and/ or can get side railed.	• *Adapting and flexing on the fly to the needs of the group or environmental changes is important.*<br>• *Come prepared with a backup plan especially if technology is being used.*<br>• *Prioritize the agenda so that you take care of the items that are most important first.*
**Group ideas are superficial**	Groups may not share the deepest and/or most thoughtful ideas because they may not understand the question or may not feel brave to do so.	• *"Anything else": Asking the group to list out other examples or solutions often gets at the more creative, obscure ideas. This moves beyond the more obvious answers.*<br>• *"What else may be a solution? Let's think about some really out of the box ideas."*<br>• *"Why else might she be feeling this way?"*
