# Supplementary material for: The Strength of a Story: Partnering With a Community Organization to Destigmatize Substance Use Disorder
Source: MedEdPORTAL. 2025 Jan 24;21:11487. doi: 10.15766/mep_2374-8265.11487 (PMC11759220; doi:10.15766/mep_2374-8265.11487)
Supplement: Supplementary file 1 — Faculty Facilitation Skills Handout.pdfSession Guide.docxPostsession Survey.docx [file mep_2374-8265.11487-s001.zip › _Educational Summary Report_11487.pdf]

MedEdPORTAL® The AAMC Journal of Teaching and Learning Resources

Original Publication Open Access

The Strength of a Story: Partnering With a Community Organization to Destigmatize Substance Use Disorder

Noah Sorkow, Cameron Lauf, Stephen H. Berns, MD*

*Corresponding author: stephen.berns@med.uvm.edu

Abstract

Introduction: Stigmatizing attitudes held by health care professionals against individuals with substance use disorder (SUD) result in worse clinical outcomes. Story-listening has been shown to help mitigate bias for medical trainees. We created a narrative-based small-group facilitated discussion between medical students and an individual in recovery from SUD through a direct partnership with a community peer-recovery organization. **Methods:** All session materials were formulated in direct partnership with the community organization. After completing prework, second-year medical students engaged in a 1.5-hour facilitated discussion with a community member in recovery and one attending physician preceptor. Student perceptions of the session and SUD were evaluated using open-ended and Likert-scale questions through an online survey. Community members engaged in their own postsession debrief. **Results:** One hundred twenty-four second-year medical students, 16 attending physician preceptors, and 10 community members in recovery participated in the session. Students agreed or strongly agreed that they appreciated the session format (92%), that they learned something new about SUD (83%), and that the session was applicable to their future career (92%). Students appreciated the small-group format and hearing someone's lived experience/perspective. Community members expressed how the session helped humanize health care providers and had interest in participating in future sessions. **Discussion:** Working in direct partnership with a local community organization to create an intimate narrative-based educational experience for medical students is feasible, appreciated by medical student participants, and mutually beneficial to community members and can facilitate positive changes in student knowledge and, potentially, bias regarding SUD.

Keywords
Community Partnerships, Patient Narratives, Small-Group Discussions, Substance Use Disorder, Case-Based Learning, Pain Medicine, Reflection/Narrative Medicine, Substance Abuse/Addiction, Community-Engaged Learning, Community Engagement

Educational Objectives

By the end of this activity, learners will be able to:

1. Recognize how stigmas and a health care provider's interactions can both help and harm those with substance use disorder.
2. Practice perspective taking to better understand the disease of addiction.
3. Engage in compassionate listening and self-reflect on their own biases, assumptions, and privileges within the context of caring for persons with substance use disorder.
4. Name inclusive strategies such as language choice in advocating for a patient with substance use disorder.

Citation:
Sorkow N, Lauf C, Berns SH. The strength of a story: partnering with a community organization to destigmatize substance use disorder. *MedEdPORTAL*. 2025;21:11487.
https://doi.org/10.15766/mep_2374-8265.11487

Introduction

In late 2022, the Centers for Disease Control and Prevention reported that the average life expectancy in the United States fell for the second year in a row.[1] The report suggested that the primary drivers for this drop in life expectancy were COVID-19 and drug overdoses, most notably, synthetic opioids. Rates of substance use for Americans of all genders, races, and ethnicities have continued to rise in the past few years,[2] particularly due to COVID-19, when increased rates of overdose were related to depression, stress, financial strains, and isolation.[3] The continued rise of opioid-related deaths and substance use disorder (SUD) has impacted families, communities, and the health system, costing 442 billion dollars annually.[4] There are complex and deeply rooted reasons for the rise in substance use and, specifically, the opioid epidemic, including stigma for those with opioid use disorder (OUD). Stigma is defined as a process where people with a particular social identity are labeled, stereotyped, and devalued, leading to discriminatory behavior

Copyright © 2025 Sorkow et al. This is an open-access publication distributed under the terms of the Creative Commons Attribution license.

against them.[5,6] Public stigma has been shown to be a significant barrier to SUD treatment, leading to lower rates of treatment seeking and poorer treatment outcomes.[7] A participant in a focus group around SUD stigma shared, “My son would not reach out for treatment… [He] just didn’t want to be thought of that way…. I tried to get him to consider treatment and he would not because of the stigma.”[8] Studies have found that stigma may also lead to decreased self-esteem, fear of punishment, and worsening overall physical and mental health.[9] Health care workers still have stigmatizing attitudes and biases towards persons with SUD,[10-12] leading to worse clinical outcomes, including delay in treatments for SUD and effective pain management.[13,14] Because of this health care crisis, there has been a call to better educate our trainees about SUDs with an emphasis on mitigating bias.[15-17]

Several educational methods have been shown to reduce stigma among trainees, including clinical experiences,[18] patient panels,[19,20] and online trainings.[21] Narrative and contact-based education has been shown to have a significant impact on building compassion, increasing empathy, and reducing emotional exhaustion and stigma.[22,23] For OUD, a recent study that combined visual campaigns about nonstigmatizing language and a narrative vignette featuring the voices of people with OUD had more impact than visual campaigns alone in reducing stigma.[24] Interestingly, Mort, Díaz, and Beverly demonstrated that medical students who experienced a five-member panel of opioid content experts and an individual in recovery had no difference in OUD stigma scores compared to students who attended lectures on OUD treatment.[25] They hypothesized that the narrative presented in the panel concentrated more on career-related stories as opposed to personal experiences.

Involving community partners to help shape educational activities with a focus on personal narratives can help provide perspectives that are unique and impactful. When instructing trainees on the social determinants of health, including community organizations has been recommended.[26] It is also apparent that involving community organizations in medical education is appreciated by students and is mutually beneficial to the community partner in helping them advance their mission.[27,28] There is, however, little evidence on whether a direct community partnership and hearing patient stories in an intimate small-group setting would be feasible, be appreciated by learners, and change the perceptions of both the students and the guests sharing their narratives. We at the Robert Larner, M.D., College of Medicine at the University of Vermont (LCOM) partnered with a community organization for persons in recovery from SUD to help guide the content of an intimate storytelling experience. Together, our aim was to assess the feasibility and acceptability of a workshop focusing on personal narratives of individuals with SUD, with a secondary aim of increasing awareness of and changing perceptions about SUD in medical trainees.

Methods

Curriculum

The curriculum at LCOM included a longitudinal course called Professionalism, Communication, and Reflection (PCR) that aimed to develop professionalism, interpersonal skills/communication, and self-assessment/reflection. This course was delivered to medical students in a consistent process-oriented discussion group with a physician preceptor from first year through the end of third year. Physician preceptors represented a variety of specialties and were all provided with facilitation skills training prior to the start of the course (Appendix A). PCR had five sequential themes building off one another and tracking alongside clinical exposure. Our session was placed in the Advocacy in Medicine theme completed by preclinical second-year medical students. This timing allowed students to be exposed to perspectives and narratives regarding addiction and SUD prior to their exposure to the hidden curriculum of the clinical environment.

Students shared that the previous year’s Substance Abuse and Addiction session, which had consisted of reading and discussion, felt disengaging and distant from the actual opioid crisis in Vermont. To improve upon the past session, we partnered with the Turning Point Center of Chittenden County—a peer-recovery support organization—to cocreate a new session that would deliver local community perspective and lived experiences.

Through this partnership, we developed a series of 10 questions meant to elicit a speaker’s experience with SUD and, specifically, any instances in which they had experienced bias on behalf of health care providers (Appendix B). These questions were refined based on the input of second-year medical students to assess for any student-perspective gaps. The questions were used to create a facilitation guide that was distributed in advance to students, faculty, and community guest speakers (Appendix B). The required set of 10 questions listed in the facilitation guide covered introductions, the guest’s substance use narrative, and stigma/bias they had experienced while interfacing with health care. This helped ensure standardization across all PCR groups.

Prework: Second-year medical students were given two prework assignments (a TED Talk and a *New York Times* article) to

Copyright © 2025 Sorkow et al. This is an open-access publication distributed under the terms of the Creative Commons Attribution license.

introduce the social factors confounding the development and treatment of SUD. Prework did not cover the pathophysiology of addiction and treatment as this was covered elsewhere in the medical school curriculum. The TED Talk, *Everything You Think You Know About Addiction Is Wrong,* detailed the socioeconomic and psychosocial factors behind addiction.[29] The *New York Times* article, “It’s Misleading to Call Addiction a Disease,” was an op-ed written by a physician with SUD that argued whether or not addiction should be considered a disease.[30] This prework was selected directly with representatives from the Turning Point Center. As a substitute for these prereadings, we would recommend an article entitled “Language Matters: It Is Time We Change How We Talk About Addiction and Its Treatment,” which details evolving perceptions on discussing SUDs.[31]

Session structure: Each PCR group (six to eight students) and its physician preceptor were paired with a guest speaker in recovery from SUD from the Turning Point Center. Speakers were provided with a list of the students in each PCR group a week before the session to avoid personal or privacy conflicts. In the hour before to the sessions, all guest speakers underwent an in-person briefing covering an introduction to PCR, where the students were in their medical training, and the general format of the session.

The 90-minute session was student led, with a physician preceptor/facilitator as a guide to ensure quality, consistency, and adherence to the written guide (Appendix B). Each session began with a 10-minute warm-up exercise where students brainstormed and shared reasons why someone in recovery and someone actively using might feel uncomfortable seeing a physician. Afterwards, the facilitator reminded the students of their community agreement, including confidentiality. The session then transitioned to question and answer with the Turning Point Center guest using the session guide. After the required questions had been asked, the session was opened for students and the physician preceptor to explore any additional questions about SUD with their guest. The speaker then left, and the session closed with the group sharing their takeaways.

Postsession debrief with guest speakers: A separate debrief was held for the guests after the session. This was a semistructured group session facilitated by the course director while the principal investigator took notes. Guests were asked what they felt had gone well, what they would like to change, and if anything came up for them during their session. After these questions, the debrief was opened to any other additional comments and concluded with gratitude for the guests.

Evaluation

A survey was developed in direct collaboration with the Turning Point Center to assess feasibility of future iterations of this session and whether learning objectives had been met. An anonymous REDCap survey was distributed through email to the second-year medical student class. The survey featured 10 questions total, including free-response items, 5-point Likert-scale items (1 = *strongly disagree,* 5 = *strongly agree*), and an optional space for additional comments. Questions were aimed at identifying whether students appreciated the session format, if they had learned new information, if their perceptions had changed, and what they liked/disliked about the session (Appendix C). Survey responses were gathered and tabulated using Microsoft Excel.

The free-response questions were analyzed using a qualitative review of the commentary to identify and report common themes among responses. Both principal investigators independently reviewed responses and coded them to one or multiple themes. After this, the coded themes identified by each investigator were cross-compared to identify which of them were agreed upon. A descriptive analysis was done on each of the seven Likert-scale questions. Themes were also identified from the debrief sessions with community members through a discussion between the community partner lead and the principal investigators.

Feasibility was defined as whether or not we were able to successfully identify a community organization that would be willing to participate, form a partnership, design our facilitation guide with community input, recruit a sufficient number of community partners to engage with each group of students, and have at least 90% student participation. As such, data were collected on student attendance and numbers of community guest speakers who participated.

Results

Participants

All 124 second-year medical students from LCOM attended the session, along with 16 physician preceptors. Ten individuals in recovery recruited by the Turning Point Center participated, eight of whom participated in both sessions, allowing for one guest per PCR group. All guest speakers attended the pre- and postsession debriefs.

Feedback From Community Guests

Overall, there was consensus that guests enjoyed participating in this program, although it made three of them nervous. The individuals who were nervous attributed this feeling to social anxiety and fears of public speaking. They reported that the

Copyright © 2025 Sorkow et al. This is an open-access publication distributed under the terms of the Creative Commons Attribution license.

anxiety diminished after interacting with the medical students and noticing that the students were interested in this topic. Multiple community members cited that this interaction humanized medical professionals in their eyes. For example, one community member in recovery stated that she was “surprised” by how easily she got along with the students and that she could see these individuals as future friends—a feeling she had never previously associated with medical professionals. Interestingly, one community member stated that participating in this session provided her insight into why her primary care provider asked her certain health screening questions during routine visits. The guest speakers expressed their desire to continue to participate in future iterations of this program, to help refine the prelearning materials to emphasize the socioeconomic drivers of SUD more adequately, and to further decrease the formality of the session to make the content more approachable to students who might be uncomfortable with this sensitive subject.

Feedback From Students

A postsession RedCAP survey was distributed to all 124 student attendees; the response rate was 45%.

The first two survey questions pertained to student opinions of the session’s format (i.e., a facilitated question-and-answer session with a community member in recovery) as shown in Figure 1. Ninety-two percent of respondents agreed or strongly agreed with the statement that they appreciated the session format, with 67% strongly agreeing. Eighty-three percent of respondents agreed or strongly agreed with the statement that they wanted more sessions similar to this experience in the future.

The next two survey questions assessed students’ perceptions on a change in their knowledge about SUD and their own biases (Figure 2). Eighty-three percent of respondents agreed or strongly agreed that they learned something new about SUD because of the session. Fifty-three percent respondents agreed or strongly agreed that their perceptions about individuals with SUD were changed because of the session, while another 28% were neutral regarding their perception changing.

Ninety-two percent of students agreed or strongly agreed when asked about the session’s applicability to their future career in medicine.

Seventy-six percent of students agreed or strongly agreed with the statement that they now recognized opportunities to get involved with volunteering and advocacy programs within the SUD community.

Answers to three free-response questions regarding what was appreciated about the session (Table 1) and what students wanted to improve upon (Table 2) are tabulated by theme, frequency of themes, and exemplary responses.

Only two students provided optional additional comments. The first student enjoyed the TED Talk provided as part of the

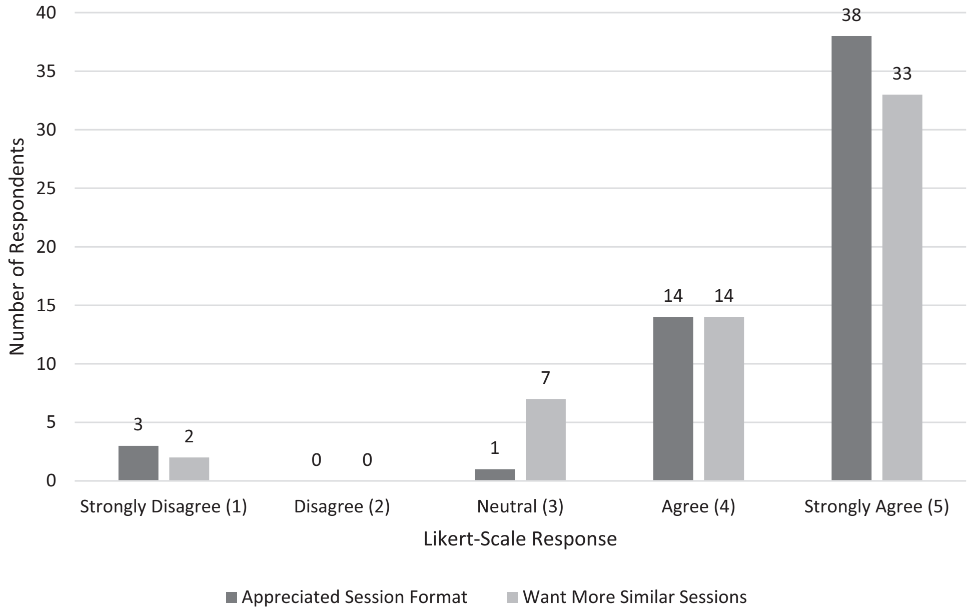

Figure 1. Numbers of students who self-rated on a 5-point Likert scale their appreciation for the session format and their desire to have similar sessions in the future.

Copyright © 2025 Sorkow et al. This is an open-access publication distributed under the terms of the Creative Commons Attribution license.

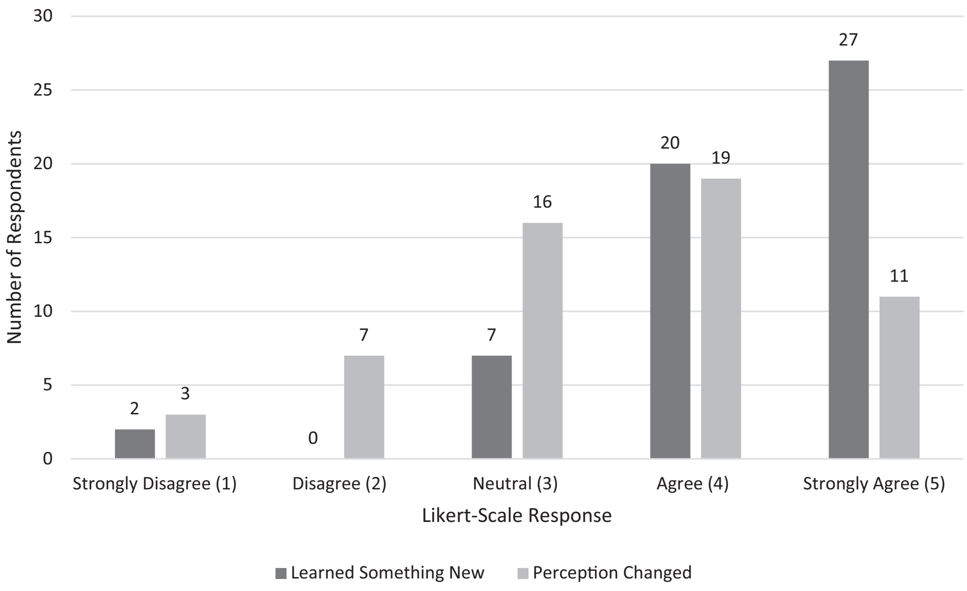

Figure 2. Numbers of students who self-rated on a 5-point Likert scale their changes in knowledge and perception.

prelearning materials. The second student thanked the team for working on improving PCR in the preclinical years.

Discussion

Our curriculum on substance use and stigma successfully demonstrated a feasible model for collaborating with a community organization to ensure an intimate, story-listening experience for medical students. Our community partner successfully recruited a sufficient number of persons in recovery to accommodate each small group and directly collaborated in all steps of curriculum development. Next, students shared their strong appreciation for the intimate session format and ability to hear a person's lived experience. They shared that this allowed for discourse that challenged misconceptions and biases and that they gained new perspectives and insights into SUD. One student commented that it even changed the perspective of their physician preceptor. Students felt that this experience was applicable to their future no matter their desired specialty. Community members remarked that this was an overall positive experience in contrast to their past interactions with the health care system. The community guests shared that this experience humanized medical professionals and provided insight on why clinicians choose the questions they ask of their patient, for example. Community guests shared enthusiasm for wanting to repeat the session the following year. While not directly one of the aims of the curriculum, it

Table 1. What Students Appreciated About the Session

Theme	Prevalence in Responses	Representative Responses
Opportunity to speak to a person with lived experience	80%	"Being able to speak to someone who has experienced substance abuse was much more helpful than I think us discussing substance abuse on our own. I found it really valuable to ask [the speaker] direct questions for both how I approach people suffering from these problems both in my personal life and in my future role as a doctor."<br>"Really putting name and face to the condition and challenges and was an incredible privilege to meet with someone."
Session format	18%	"I loved how we had an individual from the community to ask questions to. It was my favorite PCR session so far."<br>"It is just great to speak with and ask questions to someone who has real life experience on substance use disorder, instead of talking about theoretical all the time! Very powerful session."
Learning from perspectives outside of traditional medical education	12%	"[The speaker's] perspective, having lived through the challenges of substance use, was invaluable to bettering our understanding of how to effectively and respectively care for patients."<br>"I loved hearing stories and getting a new perspective on substance use disorder. I think our session was great and we really got a more detailed look into our faculty member's perspectives. She even challenged some of the thinking of our PCR mentor which was great to see."

Abbreviation: PCR, Professionalism, Communication, and Reflection.

Copyright © 2025 Sorkow et al. This is an open-access publication distributed under the terms of the Creative Commons Attribution license.

Table 2. What Students Thought Was Missing From the Session

Theme	Prevalence in Responses	Representative Responses
Responded "N/A"	78%	N/A
Actionable next steps to get involved in advocacy work	7%	"I think we could discuss more about how we as doctors can best help patients with substance use disorder, both in clinic/in the hospital and also as community advocates."<br>"More of what we as providers can do."
More variety guest speaker narratives	5%	"I think the varying SUD meant that they could not provide information first hand about opioids, alcohol, etc."<br>"Our guest did not have experience with traditionally addictive substances and quit her substance without interacting with the medical field. It would have been nice to have the opportunity to speak with someone who struggled more with sobriety so that we could understand how that tension plays out."<br>"It might have been cool to read a different story of someone's experience to provide for discussion after the guest left."
More in-depth prereading	2%	"Prereading could have been more in depth."
Session-specific facilitators	2%	"I think this might have been a good session to have a faculty member facilitate. Not necessarily our PCR instructor but someone with a little more experience. I felt that sometimes certain statements that were made were cut off in an attempt to cover all of the questions in the session guide and someone with a little more experience validating people who have gone through this might have been helpful to protect the emotions of the person sharing."
Instruction on taking a substance use history	2%	"I would have appreciated to hear both our physician's and community member's perspectives on specific questions and ways to ask about substance use in order to get someone proper medical help and social support."
Better communication of students' baseline knowledge of SUD to guest speakers	2%	"Our community member seemed to assume we had no baseline knowledge of substance use disorders (which life experience, our classes, and the pre-work had prepared us well for) and spent most of their time on introductory content. I wish they knew we already had that baseline knowledge so they could build on it and help us become better physicians (they emphasized being empathetic, but did not provide much advice in the way of how our unique role as physicians can be best used to support our patients)."
Instruction on language surrounding SUD	2%	"There was a lot of discussion around language in the pre-work, but didn't specify what patients actually prefer, and I know it's very variable, but in just talking to our guest, I wasn't really sure how to address their history of substance use without sounding overly technical."

Abbreviations: PCR, Professionalism, Communication, and Reflection; SUD, substance use disorder.

became apparent that guest speakers developed a more positive opinion of health care providers through their participation in the session. We believe that the small-group guest experience over a patient panel discussion allowed for vulnerability from both students and guests and helped change perceptions mutually.

Our emphasis on sharing the narratives of community members, although greatly appreciated by most students, left some of them feeling a lack of concrete clinical guidance. This contrasts with the Mort, Díaz, and Beverly study, which emphasized professional stories.[25] Furthermore, based on student feedback, it became clear that while this curriculum provided in-depth exposure to a person's lived experience, it came at the expense of diverse narratives. A panel format can better preserve variety in narratives but may sacrifice intimacy and engagement. Alternatively, an additional written or recorded narrative from another community member could be added to the session materials.

There was initial concern that the strong relationships within each PCR group would present a barrier to community guest speakers feeling welcome and comfortable in sharing their story. However, speakers remarked that they felt immediately welcomed into this setting.

Our activity has multiple limitations. First, it was conducted at a single site and in a state known for its smaller population and hub-and-spoke model for substance use treatment. These factors offered more favorable conditions for creating community partnerships. Additionally, our cohort of 124 students was logistically easier to accommodate with guest speakers while maintaining the small-group format. Larger schools may have to recruit more individuals from outside their immediate local community partners or utilize a virtual format if they have multiple campuses or distant community partners. As the primary endpoint was to establish feasibility for the session, a presurvey was not conducted in an attempt to reduce medical student survey fatigue. Because of this, we were unable to measure the magnitude or direction of perception and stigma with postsurveys alone. Third, only 45% of students completed the survey, which may not have been representative of the entire class perspective. This relatively low rate of survey completion can likely be attributed to delayed survey distribution after the date of the session. Finally, we must mention that the existing curricular framework for PCR was conducive to our implementation of these sessions. Specifically, resources at our disposal included preformed small groups of medical students, preestablished longitudinal intragroup relationships, dedicated time within the medical school curriculum, dedicated physical space, and

Copyright © 2025 Sorkow et al. This is an open-access publication distributed under the terms of the Creative Commons Attribution license.

preassigned faculty preceptors who had already received facilitation training. Given these resources, there are no plans to separate this session from the larger PCR curriculum. However, these resources are not unique to PCR, and this activity can be implemented as a stand-alone session by adding presession time for faculty and guests to meet and review the session guide and facilitation skills (Appendices A and B). For programs without longitudinal small groups, it would be beneficial to spend time at the beginning of the session to create and reinforce a community agreement.

To further improve the curriculum, we plan on increasing the diversity of narratives via video vignettes to be included in the prework, create dedicated faculty development time regarding SUD language and sensitivity, and reserve 10 minutes at the end of each session for specific conversation about what can be done from the provider perspective.

To better assess the impact of the future sessions, we plan on conducting pre- and postsession surveys of students using a validated instrument that measures explicit bias regarding SUD and distributing the surveys during the session. This would better assess the session's efficacy in regard to the stated learning objectives and improve response rates.

For students who desire further involvement with this community, we plan to partner with the Turning Point Center to create a volunteer event for the creation of substance use recovery kits and their distribution.

Appendices

A. Faculty Facilitation Skills Handout.pdf

B. Session Guide.docx

C. Postsession Survey.docx

All appendices are peer reviewed as integral parts of the Original Publication.

Noah Sorkow: Fourth-Year Medical Student, Robert Larner, M.D., College of Medicine at the University of Vermont

Cameron Lauf: Executive Director, Turning Point Center of Chittenden County

Stephen H. Berns, MD: Associate Professor, Division of Palliative Medicine, Department of Family Medicine, Robert Larner, M.D., College of Medicine at the University of Vermont; ORCID: https://orcid.org/0009-0001-0734-1794

Disclosures
None to report.

Funding/Support
None to report.

Ethical Approval
Reported as not applicable.

References

1. Xu J, Murphy SL, Kochanek KD, Arias E. *Mortality in the United States, 2021*. National Center for Health Statistics; 2022. NCHS Data Brief 456. Accessed December 30, 2024. https://doi.org/10.15620/cdc:122516
2. Spencer MR, Miniño AM, Warner M. *Drug Overdose Deaths in the United States, 2001–2021*. National Center for Health Statistics; 2022. NCHS Data Brief 457. Accessed December 30, 2024. https://doi.org/10.15620/cdc:122556
3. Tanz LJ, Dinwiddie AT, Snodgrass S, O'Donnell J, Mattson CL, Davis NL. *A Qualitative Assessment of Circumstances Surrounding Drug Overdose Deaths During the Early Stages of the COVID-19 Pandemic.* Centers for Disease Control and Prevention; 2022. SUDORS Data Brief 2. Accessed December 30, 2024. https://www.cdc.gov/overdose-prevention/media/pdfs/SUDORS-Data-Brief-2.pdf
4. Addiction and substance misuse reports and publications. U.S. Department of Health and Human Services. Updated August 31, 2023. Accessed December 30, 2024. https://www.hhs.gov/surgeongeneral/reports-and-publications/addiction-and-substance-misuse/index.html
5. Goffman E. *Stigma: Notes on the Management of Spoiled Identity*. Prentice-Hall; 1963.
6. Tsai AC, Kiang MV, Barnett ML, et al. Stigma as a fundamental hindrance to the United States opioid overdose crisis response. *PLoS Med*. 2019;16(11):e1002969. https://doi.org/10.1371/journal.pmed.1002969
7. Corrigan PW, Nieweglowski K. Stigma and the public health agenda for the opioid crisis in America. *Int J Drug Policy*. 2018;59:44-49. https://doi.org/10.1016/j.drugpo.2018.06.015
8. McCurry MK, Avery-Desmarais S, Schuler M, Tyo M, Viveiros J, Kauranen B. Perceived stigma, barriers, and facilitators experienced by members of the opioid use disorder community when seeking healthcare. *J Nurs Scholarsh*. 2023;55(3):701-710. https://doi.org/10.1111/jnu.12837
9. Latkin C, Davey-Rothwell M, Yang J, Crawford N. The relationship between drug user stigma and depression among inner-city drug users in Baltimore, MD. *J Urban Health*. 2013;90(1):147-156. https://doi.org/10.1007/s11524-012-9753-z
10. Stone EM, Kennedy-Hendricks A, Barry CL, Bachhuber MA, McGinty EE. The role of stigma in U.S. primary care physicians'

Copyright © 2025 Sorkow et al. This is an open-access publication distributed under the terms of the Creative Commons Attribution license.

treatment of opioid use disorder. *Drug Alcohol Depend*. 2021; 221:108627. https://doi.org/10.1016/j.drugalcdep.2021.108627

11. Hall EA, Cernasev A, Nasritdinova U, Veve MP, Hohmeier KC. Stigma of opioid use disorder and its indirect effects on student pharmacists' perceptions and attitudes. *Pharmacy (Basel)*. 2020;8(3):144. https://doi.org/10.3390/pharmacy8030144
12. Wimberly AS, Petros R, Sacco P, Tennor M, Murray-Browne S, Arikat L. Factors that contribute to stigma toward opioid users among social work students. *J Soc Work Pract Addict*. 2024; 24(1):37-48. https://doi.org/10.1080/1533256X.2022.2103982
13. Harsanyi H, Cuthbert C, Schulte F. The stigma surrounding opioid use as a barrier to cancer-pain management: an overview of experiences with fear, shame, and poorly controlled pain in the context of advanced cancer. *Curr Oncol*. 2023;30(6):5835-5848. https://doi.org/10.3390/curroncol30060437
14. Isbell LM, Chimowitz H, Huff NR, Liu G, Kimball E, Boudreaux E. A qualitative study of emergency physicians' and nurses' experiences caring for patients with psychiatric conditions and/or substance use disorders. *Ann Emerg Med*. 2023;81(6):715-727. https://doi.org/10.1016/j.annemergmed.2022.10.014
15. Ram A, Chisolm MS. The time is now: improving substance abuse training in medical schools. *Acad Psychiatry*. 2016;40(3): 454-460. https://doi.org/10.1007/s40596-015-0314-0
16. Ratycz MC, Papadimos TJ, Vanderbilt AA. Addressing the growing opioid and heroin abuse epidemic: a call for medical school curricula. *Med Educ Online*. 2018;23(1):1466574. https://doi.org/10.1080/10872981.2018.1466574
17. Schleiter K, Johnson L, Combes JR. GME Stakeholders Congress on Preparing Residents and Fellows to Manage Pain and Substance Use Disorder. *J Grad Med Educ*. 2021;13(5):739-742. https://doi.org/10.4300/JGME-D-21-00792.1
18. Liu E, Moumen M, Goforth J, et al. Characterizing the impact of clinical exposure to patients with opioid use disorder on medical students' perceptions of stigma and patient care. *Teach Learn Med*. 2023;35(2):128-142. https://doi.org/10.1080/10401334.2022.2038175
19. Monteiro K, Dumenco L, Collins S, et al. Substance use disorder training workshop for future interprofessional health care providers. *MedEdPORTAL*. 2017;13:10576. https://doi.org/10.15766/mep_2374-8265.10576
20. Dumenco L, Monteiro K, Collins S, et al. A qualitative analysis of interprofessional students' perceptions toward patients with opioid use disorder after a patient panel experience. *Subst Abus*. 2019;40(2):125-131. https://doi.org/10.1080/08897077.2018.1546262
21. Bascou NA, Haslund-Gourley B, Amber-Monta K, et al. Reducing the stigma surrounding opioid use disorder: evaluating an opioid overdose prevention training program applied to a diverse population. *Harm Reduct J*. 2022;19:5. https://doi.org/10.1186/s12954-022-00589-6
22. Winkel AF, Feldman N, Moss H, Jakalow H, Simon J, Blank S. Narrative medicine workshops for obstetrics and gynecology residents and association with burnout measures. *Obstet Gynecol*. 2016;128(4)(suppl):27S-33S. https://doi.org/10.1097/AOG.0000000000001619
23. Schoonover KL, Hall-Flavin D, Whitford K, Lussier M, Essary A, Lapid MI. Impact of poetry on empathy and professional burnout of health-care workers: a systematic review. *J Palliat Care*. 2020; 35(2):127-132. https://doi.org/10.1177/0825859719865545
24. Kennedy-Hendricks A, McGinty EE, Summers A, Krenn S, Fingerhood MI, Barry CL. Effect of exposure to visual campaigns and narrative vignettes on addiction stigma among health care professionals: a randomized clinical trial. *JAMA Netw Open*. 2022;5(2):e2146971. https://doi.org/10.1001/jamanetworkopen.2021.46971
25. Mort SC, Díaz SR, Beverly EA. Using contact-based education to destigmatize opioid use disorder among medical students. *Teach Learn Med*. 2021;33(2):196-209. https://doi.org/10.1080/10401334.2020.1820869
26. National Academies of Sciences, Engineering, and Medicine. *A Framework for Educating Health Professionals to Address the Social Determinants of Health*. National Academies Press; 2016. Accessed December 30, 2024. https://doi.org/10.17226/21923
27. Kutscher E, Boutin-Foster C. Community Perspectives in Medicine: elective for first-year medical students. *MedEdPORTAL*. 2016;12:10501. https://doi.org/10.15766/mep_2374-8265.10501
28. Palakshappa D, Denizard-Thompson N, Puccinelli-Ortega N, Brooks A, Damman A, Miller DP Jr. The experiences of community organizations partnering with a medical school to improve students' understanding of the social determinants of health: a qualitative study. *Med Teach*. 2022;44(11):1260-1267. https://doi.org/10.1080/0142159X.2022.2056007
29. Hari J. *Everything You Think You Know About Addiction is Wrong*. TED. June 2015. Accessed December 30, 2024. https://www.ted.com/talks/johann_hari_everything_you_think_you_know_about_addiction_is_wrong
30. Fisher CE. It's misleading to call addiction a disease. *New York Times*. January 15, 2022. Accessed December 30, 2024. https://www.nytimes.com/2022/01/15/opinion/addiction-disease.html
31. Zgierska AE, Miller MM, Rabago DP, et al. Language matters: it is time we change how we talk about addiction and its treatment. *J Addict Med*. 2021;15(1):10-12. https://doi.org/10.1097/ADM.0000000000000674

Received: February 14, 2024
Accepted: October 16, 2024
Published: January 24, 2025

Copyright © 2025 Sorkow et al. This is an open-access publication distributed under the terms of the Creative Commons Attribution license.
